# Reply to Prof. Joynes’ Paper on “Quarantine by the General Government,” and to Prof. Chaille on “Castration and Spaying as a Means of Arresting Syphilis”

**Published:** 1880-02-20

**Authors:** Alban S. Payne

**Affiliations:** Professor of Practice, in Southern Medical College


### Southern Medical Record.

EDITORS:

T. S. POWELL, M.D.   W. T. GOLDSMITH, M.D.   R. C. WORD, M.D.

*R. C. WORD, M.D., Managing Editor.*

☞All Communications and Letters on Business connected with the RECORD must be addressed to the Managing Editor.

VOL. X.   ATLANTA, GA., FEBRUARY 20, 1880.   NO. 2.

### ORIGINAL AND SELECTED ARTICLES.

### *REPLY TO PROF. LEVIN S. JOYNES' PAPER ON "QUARANTINE BY THE GENERAL GOVERNMENT," AND TO PROF. CHAILLE ON "CASTRATION AND SPAYING AS A MEANS OF ARRESTING SYPHILIS."*

BY DR. ALBAN S. PAYNE,

Professor of Practice, in *Southern Medical College.*

State medicine, preventive medicine, I regard to be that branch of knowledge which treats of the general means to be employed under public authority for the preservation of the public health—in other words, it is public hygiene. It is as old as the Pentateuch, which embraces many of its provisions, and has engaged the serious attention of medical men from the most remote period to the present time. Hygiene is defined to be health, or the art or science of preserving health, that department of medicine that treats of the preservation of health. This is the true definition of "State Medicine," in its proper and most restricted sense; but the word state is susceptible of a very wide signification. Joined to another word it denotes public, as "state affairs," "state policy," etc., etc.; any body of men, united by profession, or constituting a part of the integral portion of the people.

Medicine, in the largest sense of the term, comprehends everything pertaining to the knowledge, cure and palliation of diseases. It also embraces the alleviation of pain and the prevention of disease in the inhabitant or inhabitants of a country. State medicine, then, in its broadest signification (the manner in which I propose to discuss it), may be likewise defined : The art of preventing, alleviating and curring the diseases of the inhabitants of the State, under the dtrection of public authority.

In this wide range it will be seen to embrace all our public charities: Our asylums for the protection and cure of the insane; the asylums for the instruction and maintenance of the deaf and dumb, and the blind; our inebriate asylums, for the relief and cure of the unfortunate victim to alcoholic indulgencies; our alms houses; our hospitals, jails, infirmaries and dispensaries, etc. To illustrate this position we will take a single example. We suppose, for instance, that from the excessive use of alcoholic stimulants the poor unfortunate victim of intemperance has so unsettled his nerves that he finds himself wholly powerless to resist his *mania* for strong drink; at the same time he possesses the most painful knowledge that by a continued gratification of his depraved appetite, he is being dragged down day by day deeper and deeper into the slimy vortex of everlasting shame and degradation. In this sad state of affairs his friends, or the public authorities, send him to an inebriate asylum, where he receives the proper medication and care. His health is restored to its pristine vigor, his mania is gone, he is a rational being once more.

"Richard is himself again."

This is unquestionably State preventive medicine.

Again, disease produces disease—like begets like, death begets death. And be it an act, or a word, or a single dose of medicine, so it resists, death, suffering or disease, in a single individual or in a whole community, it becomes, *de facto* and *de jure*, preventive medicine. I do not doubt that a kindly word opportunely spoken, a moral lecture well delivered, has often proven to be preventive medicine. It has proven so by changing the determination of some one who has contemplated in the silent chambers of his poor, sad heart, that awful act of self-destruction, a sentiment much oftener entertained in the human bosom than is generally supposed to be the case. Many a useful man now living who is an ornament to the circle of society in which he moves, has, at some period of his life, entertained some such ideas. Had not this determination been changed, it may be by the kindly word, or by the moral lecture, and the terrible act been committed, the result would be that in a few short months or years, we should have seen a fond darling mother, a proud father, an affectionate brother or the darling sister, with all the bloom and beauty on her life like features, "fall as the leaves fall" into the cold, silent grave dug beneath the shade of the wide spreading "weeping willow," here to rest until the grand day of the final resurrection. Has not this kind word, this moral lecture, proven itself to be *preventive medicine?* When we consider the great good accomplished by "state medicine" in the last 30 years, we shall find much to praise and to honor in those noble, self-sacrificing men who have controlled our public Institutions during this long period of years; and who have brought them to that state of perfection so creditable to the present age. This should encourage us to press on in the future, until the working of our institutions shall be equal to those of any nation in the world, and shall reach that state of perfection so much desired by the philanthropist.

Some of these great improvements consist in the notorious fact that the buildings are much larger, roomier and much better arranged in regard to convenience and comfort. There is also much more care given to *ventilation* now than formerly. The treatment of the insane is much more humane, much more conducive to their amusement and comfort, than it was thirty years ago. The same facts hold good in regard to almost all of our State institutions.

However, for one *shocking* instance to the contrary, see Dr. C. W. Chancellor's report "On the Public Charities, Reformatories, Prisons and Alms-Houses of the State of Maryland, July, 1877."

I have said by these great achievements in the past we should be encouraged to press on in the future with this important and noble work of "preventive medicine." But at the same time we should do so modestly, carefully, composedly, sensibly, without a single thought of self-aggrandizement and entirely *untrammelled* by either fear or favor. Now, I am one of those who do not like the working of the "National Board of Health." Its proceedings seem to *smack* too much of *sensationalism* either to engage my confidence or to elicit my admiration. If it had more medical and less politico-medical jurisprudence about its proceedings, it might be different with me. Their action is too much that of a "great ring" organized for and intent on gaining the sympathetic ear of the public through the halls of Congress, than it is like that of a careful, sensible, painstaking body of scientific gentlemen calmly collecting the dry, uninteresting facts in regard to the prevention and cure of epidemic diseases. Moreover, it appears to my plain, *unvarnished* way of thinking, most all the articles I have recently read on preventive medicine seem too much as if prepared for the eye of the politician and the public, or other than the benefit of the hard-working, careful practitioner of medicine. Hence we find those sensational terms, "freezing out" and "stamping out" becoming "fashionable parlance" in medical communications, or rather in what should have been strictly medical communications.

Such sensational terms should never be used by the *regular* physician, unless in a sense of ridicule. "Freezing out" the contagia of yellow fever is too *puerile* to merit any consideration in this paper. Only I should like to know how Professor Gamgee would go about "freezing out" the contagia of yellow fever in a vessel containing a cargo of "monkeys" and sacks of coffee!

Here I presume the "freezing out" would give place to the "stamping out" process and the poor monkeys would be killed and the coffee burned and our broad-backed Uncle Sam made to foot the bill.

If this "freezing out" process was puerile in conception, the excuse given for the failure of the experiment in the vessel sent North for the purpose of testing the value of the discovery, was even more childlike; namely, that the experiment would have proved a *grand* success if the contagia of yellow fever had not found some rotten wood in the vessel and hid itself in this wood. Now, I was taught to believe the decay of vegetable matter to be a sort of "slow combustion." If this is true, it ought to prove a destroyer of contagia rather than a protector of its virus.

The manifold publications of the present day on personal and public hygiene, prove the great and increasing interest of intelligent communities in this important subject of the prevention of disease. This branch of human knowledge, to which the attention of good men has been directed in all ages, is still in its infancy. Grave topics, such as contagion and quarantine, are still discussed, with conflicting arguments often based upon partially ascertained facts, and no absolute conclusion has been arrived at to satisfy all parties.

We have had at times to lament in some of the "brochures" the evidence of insufficient acquaintance with the collateral sciences, and in the arguments used the absence of the vigorous rules of logic.

Some of these essays might, indeed, please the laymen, but would impart little information to the trained professional men.

It is a trite saying that prevention is more important than cure. When a disease is once established, its issue is doubtful and its treatment difficult.

Public hygiene has in charge the removal of the external causes of diseases.

Hitherto it has provided nothing to correct aerial conditions, such as hygrometric, temperature, ozone, electric state, barometrical perturbations, etc., etc. It has failed to elucidate the nature of traveling epidemics and modes to combat their prevalence. To avoid acrid contaminations and influences, its *sole resource* is, when the ability exists, to shift places in quest of more serene air and better localities as do the migratory birds, or at least to seek what may be at hand, shelter from heat, cold and infection. In this instance instinct is as good a teacher as reason.

Much has been said and reiterated about stagnant, impure and corrupt waters, soil saturation, vegetable, animal and mineral impregnation, of potable water, etc., etc., etc. The means recommended have been improved drainage, sewerage, water conduction, percolation, the substitution of cistern in lieu of spring or well water, thorough cultivation of the soil, etc., etc.

In every instance the corrective forces of nature have been ignored. In other words, its perpetual agency to remove impurity by land and by sea. Personal hygiene consists, in brief, in economizing the forces of the system by the maintenance of bodily cleanliness, purity of morals, proper dietetic rules, suitable clothing and the avoidance of all noxious agencies and habits.

> "Observe the rule
> Of not too much by temperance thoughts
> In what thou eatest or drinkest."—*Milton.*

Let us turn the attention to preventive medicine as applied to the person in averting any given disease. The list of means which can be so applied is unfortunately limited. As limited as it is, some advantage to future progress may be gained by considering them at this time.

*Sea Scurvy.*—This scourge of the early voyagers gave occasion to the most splendid triumph of preventive medicine on record. The chief credit is due to Capt. Cook, who, in his celebrated circumnavigation of 3 years duration, preserved the health of his entire crew of 118 men from this disease by proper regimental treatment. But one man died and he of phthisis.

*Vaccination* discovered by the immortal Jenner and still further utilized by Payne. By this great discovery, the saving of life and of dis figurement and suffering to the human family, is too immense to be estimated by figures. Millions upon millions of the human race yet unborn will reap its benefits.

*Alcohol* as a good preventive of diphtheria. The value of this discovery appears about to be realized.

*Carbonate of Ammonia*, hartshorn, as an antidote to the bite of the rattlesnake (crotalus horridus). This remedy will be found a perfect antidote to all poisonous bites and stings of all reptiles, etc., etc., whose poison is quick and prostrating in its effects upon the system of the mammalia in contra-distinction to rabies canina, the poison of which is *very* slow in its effects upon the mammalian system, and the antidote to which, when found, will prove to be, in the opinion of the writer, a depressant and acid poison. The discovery of the hartshorn being an antidote to the bite of the rattlesnake was made in 1852, by the writer.

*Belladonna.*—At times evidently preventive of scarlct fever.

*Carbolic Acid* as preventive of malaria and scarlet fever.

*Sulphite Soda.*—To some extent preventive of scarlet fever.

And here I would take occasion to bear a willing testimony to the great value of a recent paper on the etiology of "Typhoid Fever," from the pen of A. M. Fauntleroy, M.D., of Staunton, Virginia, and of whom it may be truthfully said, "Tortigit nihil quod non ornavit."

The history of the late epidemic on the Mississippi coast, has shown that no notable progress has been made in the etiology or treatment of yellow fever; in fact, we know little more than did the eminent Lining, of South Carolina, in the last century, and, notwithstanding the circular of Surgeon-General Hammond and various new departures, that have been attempted, the therapeutic recommendations of the past generation of physicians still stand the tests of experience. The only real improvement that can be alluded to consists in the appropriation of the benefits derivable from nitrate of silver in black vomit, as these benefits were first made known to the world in gastritis by our friend, the lamented Gillespie, of Louisa county, Virginia.

*Public Baths.*—Hot baths, cold baths and medicated baths. The occasional use of the hot bath is a valuable preventive as well as auxiliary to cure in the treatment of diseases of the skin. The cold bath is, where regularly used and when not contra-indicated by being disagreeable to the feelings of the patient, a valuable tonic to the general system and very beneficial in the prevention and cure of dyspepsia. The Turkish bath, as well as any other medicated baths, is useful in certain diseased conditions of the system.

*Shade Trees.*—Are valuable as a prevention of "coup de soliel" or sunstroke. This valuable preventive is, I am happy to say, receiving more attention than formerly, especially is this the case in our Southern cities. It should receive great attention wherever the sun shines and brick and mortar are used for building purposes.

*Mineral Springs—Aperient, Diuretic, Tonic, Alterative.*—Of great preventive and curative value in a vast number of diseases, particlarly so in those diseases incident to females.

*Eucalyptus Tree as a Preventive of Intermittent Fever.*—Experiments should be repeated with this tree before any correct judgment can be found as to its relative value in destroying malaria.

*Common Pine Tree as a Preventive of Intermittents.*—Its concrete juice is a valuable diuretic remedy.

[TO BE CONTINUED.]

